# Effects of a 4-Week Off-Season High-Intensity Training Program on Aerobic Performance and Sprint Endurance Ability in Adolescent Female Football Players: A Pilot Study

**DOI:** 10.3390/jfmk10040396

**Published:** 2025-10-13

**Authors:** Marco Panascì, Carlo Castagna, Vincenzo Rago, Vittoria Ferrando, Piero Ruggeri, Emanuela Faelli

**Affiliations:** 1 Department of Experimental Medicine, Section of Human Physiology, University of Genoa, 16132 Genoa, Italyemanuela.faelli@unige.it (E.F.); 2 Centro Polifunzionale di Scienze Motorie, University of Genoa, 16132 Genoa, Italy; 3 Fitness Training and Biomechanics Laboratory, Technical Department of the Italian Football Federation, 50135 Coverciano, Italy; castagnac@libero.it; 4 Department of Education and Sports Science, Pegaso Open University, 80143 Naples, Italy; 5 Department of Sports Science and Clinical Biomechanics, SDU Sport and Health Sciences Cluster (SHSC), University of Southern Denmark, 5230 Odense, Denmark; 6 Department of Theoretical and Applied Sciences, eCampus University, 22060 Novedrate, Italy; vincenzo.rago@uniecampus.it; 7 National Youth Sports Institute, Singapore 397778, Singapore

**Keywords:** team sports, soccer, physical assessment, athletic performance, high-intensity interval training, physical fitness

## Abstract

**Background**: The off-season is often characterized by a significant decrease or even a complete cessation of training. If this reduction is not planned properly, it can result in detraining. Despite numerous studies examining the effects of HIIT in football players, its specific role in mitigating detraining and maintaining aerobic and anaerobic performance during the off-season in adolescent female football players remains underexplored. Therefore, this study evaluated the effects of a 4-week off-season high-intensity training (HIIT) program on aerobic performance level and sprint endurance ability in Under-15 (U-15) female football players. **Methods**: Fifteen U-15 female football players from a professional club completed an experimental protocol consisting of two HIIT formats: Small-Sided Games (SSGs) and Repeated Sprint Training (RST), each performed twice weekly. Before and after the intervention period, participants performed the Yo-Yo Intermittent Recovery Level 1 (YYIR1) test to gauge aerobic performance and the 30-seconds sprint test to assess sprint endurance. The internal training load was monitored via heart rate (HR) and blood lactate concentration ([La]^+^), while external training load metrics included the total distance (TD), moderate-speed distance (MSD), high-speed distance (HSD), acceleration distance (≥3 m·s^−2^; ACC), and deceleration distance (≤−3 m·s^−2^; DEC). **Results**: YYIR1 improved by 57% (*p* = 0.0001; d = 1.12; 95% CI: 121.94–224.71) and the 30-s test performance increased by 13% (*p* = 0.004; d = 0.91; and 95% CI: 14.46–25.53) following the intervention period. A very large correlation between time spent at 90–95% HRmax and the 30-s test (r = 0.90, *p* = 0.0001) and YYIR1 (r = 0.81, *p* = 0.0001) performance was observed. Very large and nearly perfect correlations between DHS and YYIR1 (r = 0.82, *p* = 0.0001) and the 30-s test performance (r = 0.94, *p* = 0.0001), respectively, were found. **Conclusions**: In U-15 female football players, a four-week off-season HIIT program improved both aerobic performance and sprint endurance ability, indicating that a HIIT regime attenuates the off-season detraining, thus supporting a better-conditioned return to play. Coaches may implement 4-week high-intensity off-season programs to enhance aerobic performance and start the pre-season with a satisfactory level of aerobic fitness and sprint endurance.

## 1. Introduction

The football season is typically divided into three main phases: the pre-season (or preparatory phase), the competition phase, and the off-season or transition phase [1]. Following 10–11 months of intensive training and match play, players commonly enter a rest period lasting approximately 4–6 weeks, referred to as the off-season [2,3]. This transition phase, often seen as a period of recovery, can instead be strategically reframed as a window of opportunity to sustain or enhance key physical capacities while allowing for psychological and physiological regeneration [1].

Despite its potential, the off-season is frequently characterized by a marked reduction or complete suspension of training loads [1]. When this reduction is not adequately planned, it may lead to detraining, which is a physiological state characterized by the partial or complete loss of training-induced adaptations, including cardiovascular, metabolic, neuromuscular, and performance-related qualities [2]. Importantly, detraining is not a process in itself but a consequence of insufficient training stimuli.

The literature distinguishes between short-term detraining (up to four weeks) and long-term detraining (exceeding four weeks), each with varying degrees of impact on fitness components [4].

In youth athletes, detraining has been associated with negative changes in aerobic capacity, metabolic efficiency, muscle function, and endurance performance [5,6]. In both male and female football players, the reduction in training loads during the off-season has been shown to adversely affect body composition, sprint ability, muscle strength, and maximal oxygen uptake [1,2,7]. These declines may compromise readiness for the high workloads of the pre-season, increasing the risk of injury, particularly for those who have not maintained physical conditioning during the transition phase [2]. The issue is especially relevant in female players who may be more vulnerable to lower-limb injuries during periods of inadequate physical preparation [8].

To mitigate these risks, preserve athletic performance, and prevent adverse changes in physical fitness and body composition, targeted off-season training programs have been proposed [9]. Among these, high-intensity interval training (HIIT) has emerged as a time-efficient method for maintaining cardiopulmonary and neuromuscular capacities during transitional phases [1,10].

Specifically in young players, HIIT modalities such as Small-Sided Games (SSGs) and Repeated Sprint Training (RST) have proven to be effective in improving physiological responses and match-relevant performance metrics [11]. In this context, the combination of these two HIIT formats may provide complementary training stimuli, as SSGs mainly promote aerobic and neuromuscular adaptations, and RST enhances the anaerobic capacity and sprint performance [10,11,12,13]. This integrated approach may optimize overall physical conditioning in football players.

Furthermore, a recent systematic review by Stankovic and colleagues (2023) [14] indicated that, in young female team sport athletes, different HIIT protocols induce significant improvements in the VO_2_max, repeated sprint ability (RSA), change in direction speed, linear sprint speed, and explosive strength.

Despite these promising findings, research specifically addressing female adolescent football players remains limited. Most existing studies have focused on adult female or male soccer players, reducing the applicability of their findings to adolescent female players. Additionally, the off-season represents a critical period for maintaining physical performance, during which insufficient training may lead to performance declines and increased injury risk. Therefore, a pilot study in adolescent female soccer players is warranted to assess the feasibility, safety, and preliminary effects of a HIIT program, providing essential data to inform future, larger-scale, and age-specific interventions.

Therefore, the aim of this pilot study was to evaluate, in U-15 female football players, the effects induced by a 4-week off-season HIIT program, combining Small-Sided Games (SSGs) and Repeated Sprint Training (RST), on aerobic performance level and sprint endurance ability; moreover, the relationships between aerobic capacity, sprint endurance ability, and external training load parameters was investigated. We hypothesized that the combination of two HIIT regimes, such as SSGs and RST, within the weekly training program would improve aerobic fitness and sprint endurance, thereby helping to counteract the detraining effects commonly observed during the off-season.

## 2. Material and Methods

### 2.1. Sample Size

An a priori estimation of sample size was performed using total distance covered in the Yo-Yo Intermittent Recovery Test Level 1 (YYIR1) as one of our primary outcome measures [15]. Sample size was estimated using G*Power 3.1.9.7 (Heinrich-Heine-Universität Düsseldorf, Düsseldorf, Germany), applying a two-tailed paired-samples *t* test (two dependent means) with α = 0.05, power (1 − β) = 0.80, and an anticipated within-subject effect size (ES) of 0.70. The required sample size was 15 participants.

### 2.2. Participants

Fifteen adolescent female football players (6 defenders, 6 midfielders, and 3 forwards) were enrolled from a local professional team (age: 14.9 ± 0.6 years; weight: 56.5 ± 7.7 kg; and height: 163.7 ± 6.6 cm). In the 3 months before the commencement of the study, participants usually carried out 4 training sessions (lasting between 70 and 120 min) and 1 match per week. Inclusion criteria involved the following: (1) possess medical clearance for competitive sports and (2) grant regular participation in both testing and training sessions. Exclusion criteria were muscle or joint injuries, orthopedic problems, or any other contraindication within 3 months before the start of the study. Before each testing session, players were required to remain well hydrated and to refrain from strenuous exercise for at least 48 h. They were also instructed to avoid large meals and to abstain from caffeine in the 3 h before the sessions. Prior to the study, all participants were fully informed about the study aims and procedures. The experimental protocol conformed to the Code of Ethics of the World Medical Association (Declaration of Helsinki) and was approved by the Ethics Committee of the University of Genoa (protocol code: n. 2025/47 and date of approval: 16 April 2025). Written informed consent was obtained from the adolescents’ parents or guardians prior to participation.

### 2.3. Experimental Design

**Figure 1:**
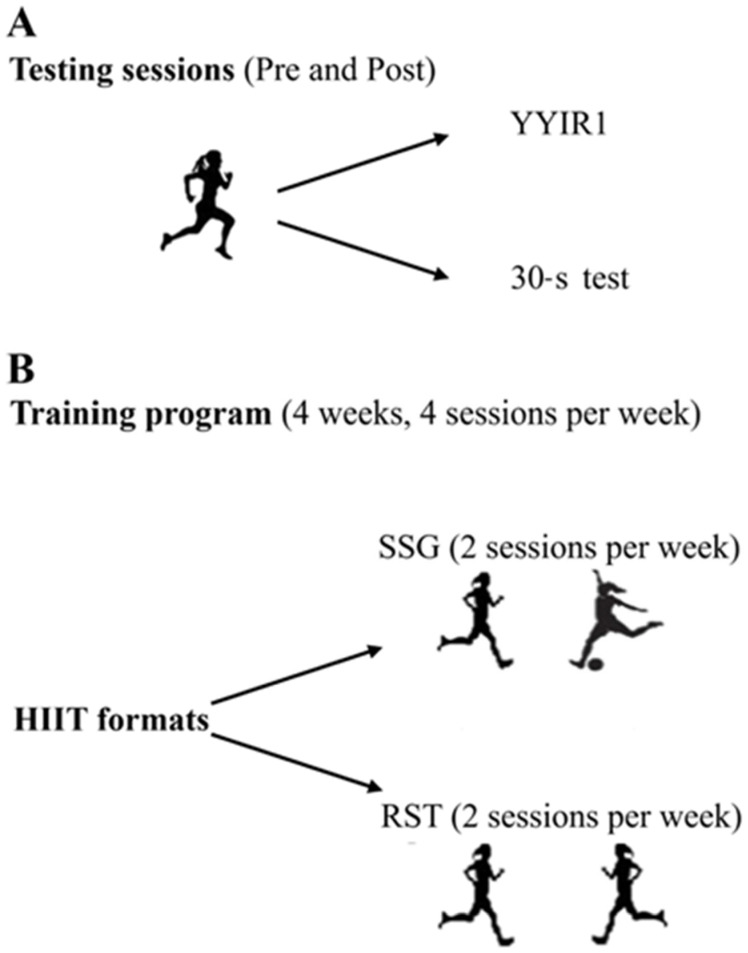
Experimental design. (**A**) Testing sessions; (**B**) training program. YYIR1, Yo-Yo Intermittent Recovery Test Level 1; 30-s test, 30-second test; HIIT, High-Intensity Interval Training; SSG, Small-Sided Games; and RST, Repeated Sprint Training.

This was a single-group, pre-post, repeated-measures study conducted during the off-season (June). After 1 month of complete training cessation, football players performed the experimental HIIT program consisting of four sessions per week (two SSGs plus two RST). Baseline (PRE) and post-intervention (POST) testing comprised the Yo-Yo Intermittent Recovery Test Level 1 (YYIR1) and the 30-s test, administered on separate days 48 h apart. All participants were familiar with both protocols before the study began (Figure 1).

### 2.4. Testing Sessions

Both the YYIR1 and the 30-s tests were conducted on a grass field under similar environmental conditions [25–26 °C, 55–60% relative humidity, and low wind (<2 m·s^−1^)] [16] and at the same time of day (15:30 ± 1 h) to minimize circadian effects [17]. Each test was preceded by a standardized warm-up consisting of 10 min of self-paced running, 2 min of skipping, 2 min of striding drills (over 10 m and 30 m, respectively), followed by 2 min of recovery. During each testing session, participants received verbal encouragement to achieve maximal effort. Throughout the testing sessions, the participants wore football boots. Each player performed a single attempt, as they were familiar with the test protocols. Both tests’ durations and distances were recorded with an electronic stopwatch (ONSTART 110, Kalenji, Villeneuve d’Ascq, France) and a GPS device (K-GPS, Montelabbate, Pesaro, Italy; 50 Hz), respectively.

Furthermore, the testing session protocols were well tolerated; all players completed every session without complications, and there was no reported dizziness, light-headedness, or nausea.

#### 2.4.1. Yo-Yo Intermittent Recovery Test Level 1 (YYIR1)

**Figure 2:**
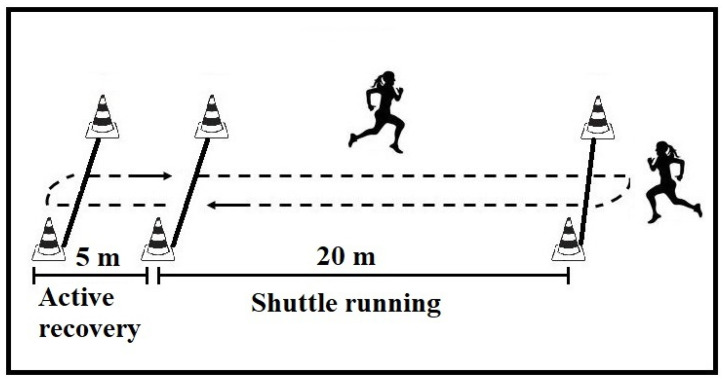
Schematic representation of the Yo-Yo Intermittent Recovery Test Level 1.

The Yo-Yo Intermittent Recovery Test Level 1 (YYIR1; Figure 2) was selected to assess high-intensity intermittent aerobic performance, and its validity and reliability for measuring aerobic performance in young female athletes has been demonstrated [18]. Specifically, YYIR1 evaluates the athlete’s capacity to perform repeated high-intensity efforts with brief recoveries, with performance expressed as total distance covered [16,19,20].

#### 2.4.2. 30-Second Test (30-s Test)

The 30-s test was chosen to assess sprint endurance ability.

Prior to this study, the preliminary phase test demonstrated good relative reliability and satisfactory absolute reliability in adolescent female players (intraclass correlation coefficient = 0.84; typical error expressed as coefficient of variation = 4.44%).

**Figure 3:**
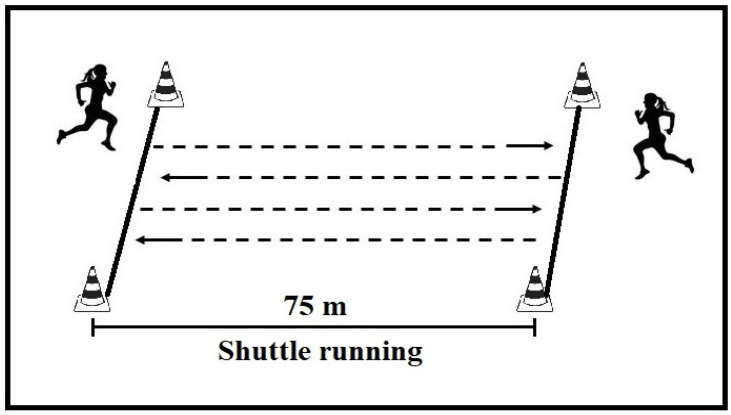
Schematic representation of 30-second test.

The test consisted of 30 s of shuttle running between two lines 75 m apart [21]. The course was delineated with disk cones placed at 1 m intervals. A whistle signaled the start and end of the 30 s run, and the total distance covered was taken as the test score. During the test, players received verbal encouragement to achieve maximal effort (Figure 3).

### 2.5. Training Program

The experimental training program consisted of 4 sessions per week (Tuesday, Wednesday, Thursday, and Friday) including 2 HIIT formats (SSGs and RST). Each format was performed twice a week. Weekly training program description is reported in Table 1. During the 4-week experimental period, players performed 16 training sessions.

**Figure 4:**
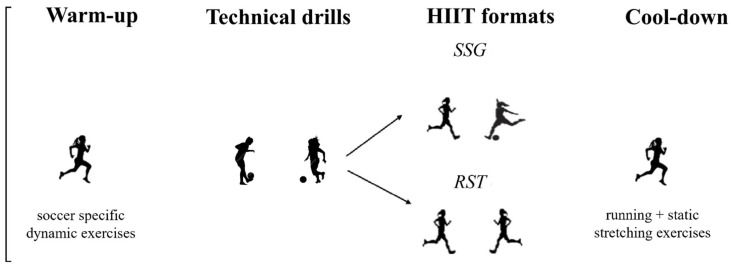
Schematic representation of training session. HIIT, High-Intensity Interval Training; SSG, Small-Sided Games; RST, Repeated Sprint Training.

Specifically, each training session lasted between 50 and 60 min and included 20 min of standardized warm-up and 10 min of technical drills, followed by SSG or RST formats and 5 min of cool-down. The total number of training sessions performed by the participants was 16. The A schematic representation of training session is reported in Figure 4.

### 2.6. HIIT Training Formats

#### 2.6.1. Small-Sided Games (SSGs)

A standardized SSG 5vs5 format with five external wildcards (EWs) positioned outside the pitch, two goalkeepers, continuous coach encouragement, and no touch limit to maximize external load was used. The pitch measured 60 × 50 m (≈300 m^2^ per player) and included two 5 × 2 m goals. Each SSG session started with a 20-minute standardized warm-up, including low-intensity running and stretching exercises, followed by 10 min of technical drills. After this, participants performed four 4-minute bouts of SSGs, each separated by 3 min of active recovery consisting of low-intensity running (self-paced). The session concluded with 5 min of cool-down including static stretching exercises.

**Figure 5:**
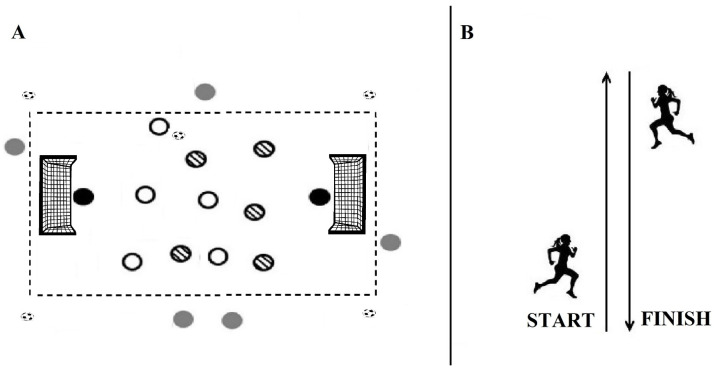
Description of both Small-Sided Games (SSGs) and Repeated Sprint Interval Training (RST) protocols. (**A**): Small-Sided Games; (**B**): Repeated Sprint Interval Training.

All standard 11-a-side rules applied, except the offside rule [22]. To minimize interruptions when the ball went out of play, spare balls were placed around the field, and four support staff were stationed pitch side to promptly return them [23]. Time lost due to major injury stoppages was excluded from the total training duration to preserve the training objectives, maximizing ball-in-play time and simulating match-specific scenarios. Throughout all SSG sessions, the intensity was monitored using GPS devices (external load) and heart-rate monitors (internal load). Description of SSG protocols is reported in Figure 5A.

#### 2.6.2. Repeated Sprint Training (RST)

RST involved 40 m (20 m + 20 m) sprints with a 180° turn [11] in all-out modality (Figure 5B). Each RST session started with a 20-minute standardized warm-up, including low-intensity running and stretching exercises, followed by 10 min of technical drills. The RST protocol was organized as follows: Weeks 1–2: 3 sets of 5 repetitions with 20 s of passive rest between repetitions and 4 min of passive rest between sets; Weeks 3–4: 3 sets of 6 repetitions with the same recovery intervals. Each session concluded with a 5-minute cool-down consisting of static stretching exercises. The intensity was monitored using GPS devices (external load) and heart-rate monitors (internal load).

### 2.7. Outcome Measures

#### 2.7.1. Physiological and Metabolic Responses

Heart rate (HR) was monitored during all experimental sessions, and HR values were recorded at 5-second intervals with an HR monitor (Polar H10, Electro, Kempele, Finland) placed inside the chest strap. Peak Heart Rate (HRpeak) was identified as the highest value recorded during each session.

Furthermore, [La]^+^ was determined 3 min after the end of each session, as previously described by Castagna et al. (2017) [24], with fingertip blood samples (5 μL), using Lactate Pro 2 (Arkray KDK, Tokyo, Japan). In particular, before each collection, according to the recommendation of the manufacturer, the finger was cleansed with alcohol and allowed to air dry [25].

#### 2.7.2. Physical Performance

Total distance values measured during both the YYIR1 (YYIR1 performance) and the 30-s test (30 s sprint performance) were chosen as indices of aerobic performance level [20,26] and sprint endurance ability [21,24], respectively.

### 2.8. Training Load Monitoring

#### 2.8.1. Internal Training Load

Internal training load was quantified from continuous heart rate (HR) monitoring throughout training session (Polar H10, Polar Electro, Kempele, Finland). Within each session, the average HR values (HRmean) were calculated, and the peak heart rate (HRpeak), defined as the highest value recorded during the session, was identified; we also derived an overall maximal heart rate (HR_max_) considered as the highest value observed across all sessions. HR time series were time-synchronized with the GPS stream for the full duration of each session. Exercise intensity distribution was expressed as time spent in five predefined zones relative to HRmax: <80%, 80–85%, 85–90%, 90–95%, and >95% HRmax [13,27].

#### 2.8.2. External Training Load

Furthermore, the external load was measured using arbitrary speed, acceleration, and deceleration categories as follows [28,29]: total distance covered (TD, m), distance at moderate speed (DMS; 14.4–19.8 km·h^−1^), distance at high speed (DHS; 19.8–25.2 km·h^−1^), distance traveled in acceleration (DACC; ≥3 m·s^−2^), and distance traveled in deceleration (DDEC; ≤−3 m·s^−2^). A portable GPS device with a sampling frequency of 50 Hz (K-sport) was used to collect data during experimental sessions. The validity and reliability of 50 Hz GPS in measuring velocity, acceleration, and deceleration during team sports has been previously demonstrated [30]. The GPS device was placed in a special pocket between the player’s shoulder blades (upper thoracic spine) in a sports vest and worn under the player’s jersey. Each device was switched on at least 15 min before each session to allow for acquisition of the satellite signal. To reduce between-device variability, each player wore the same device during each training session throughout the study.

Furthermore, the external load data were averaged weekly, based on the four training sessions performed each week.

### 2.9. Statistical Analysis

Data distribution was checked by means with the Shapiro–Wilk test and sphericity with the Mauchly test. All data were normally distributed, confirming that all parameters satisfied the assumptions for parametric analyses (*p* > 0.05).

PRE training to POST training period changes were examined with paired Student *t*-tests. Cohen’s d was used to estimate the effect size (ES), considering values > 0.8, between 0.8 and 0.5, between 0.5 and 0.2, and <0.2 as large, moderate, small, and trivial, respectively [31]. To assess how external load and peak heart rate varied over time, repeated-measures ANOVA was applied to analyze and compare the mean values of these parameters across the different weeks, with time (4 levels: 1° week, 2° week, 3° week, and 4° week) as the within-subject factor. Bonferroni post hoc tests were used in cases of significant interaction. The effect sizes for analysis of variance were reported as partial eta-squared (η^2^p), with cut-off points of 0.10, 0.25, and 0.40 representing small, medium, and high effects, respectively [31]. In addition, Cohen’s d was calculated for the pairwise comparisons between weeks to estimate the effect size (ES). According to Cohen (1988) [31], values greater than 0.8, between 0.5 and 0.8, between 0.2 and 0.5, and below 0.2 were interpreted as large, moderate, small, and trivial effects, respectively.

Furthermore, the variable association was assessed by Pearson’s product–moment correlation coefficients. Pearson values were considered: trivial r < 0.1; small 0.1 < r < 0.3; moderate 0.3 < r < 0.5; large 0.5 < r < 0.7; very large 0.7 < r < 0.9; nearly perfect r > 0.9; and perfect r = 1, as previously reported by Hopkins et al. (2009) [32]. Data were outlined as mean (SD), and the 95% confidence interval (CI) was also reported. Statistical analysis was performed using SPSS (version 20) with a significance level set at *p* < 0.05.

## 3. Results

### 3.1. Effects of Training

Results of the statistical analyses are reported in Table 2.

#### 3.1.1. Physical Performance

TD values covered during the 30-s test increased by 13% (*p* = 0.004; d = 0.91; and 95% CI: 14.46–25.53), and the YYIR1 significantly increased by 57% (*p* = 0.0001; d = 1.12; and 95% CI: 121.94–224.71).

#### 3.1.2. Physiological and Metabolic Parameters

No significant changes in the HRpeak in both the 30-s test (*p* = 0.217; d = 0.27; and 95% CI: −0.07;1.41) and YYIR1 (*p* = 0.127; d = 0.38; and 95% CI: 2.30;4.09) were found. [La]^+^ significantly increased in YYIR1 (*p* = 0.001; d = 1.37; and 95% CI: 0.99;2.45), while no changes in the 30-s test (*p* = 0.57; d = 0.14; and 95% CI: 0.15;1.19) were observed.

### 3.2. Training Monitoring Parameters

#### 3.2.1. Internal Training Load

The HRpeak did not show a significant effect of time (F(1,14) 1.187, *p* = 0.294, and pη^2^ = 0.078) during the entire training period. Pairwise comparisons revealed no significant differences between Week 1 and Week 2 (*p* = 0.06; d = 0.70; and 95% CI: −1.60 to 0.04), Week 3 (*p* = 0.09; d = 0.58; and 95% CI: −1.55 to 0.08), or Week 4 (*p* = 0.27; d = 0.51; and 95% CI: −1.44 to 0.24). Similarly, Week 2 did not differ significantly from Week 3 (*p* = 1.00; d = 0.05; and 95% CI: −0.40 to 0.54) or Week 4 (*p* = 1.00; d = 0.17; and 95% CI: −0.24 to 0.64). Finally, no significant difference was observed between Week 3 and Week 4 (*p* = 0.98; d = 0.10; and 95% CI: −0.15 to 0.41) (Table 3). The mean HR also did not show a significant effect of time (F(1,14) 3.859, *p* = 0.604, and pη^2^ = 0.020) during the training sessions. Pairwise comparisons revealed no significant difference between Week 1 and Week 2 (*p* = 1.00; d = 0.11; and 95% CI: −3.25 to 4.32), Week 3 (*p* = 0.54; d = 0.55; and 95% CI: −1.82 to 7.15), or Week 4 (*p* = 0.91; d = 0.47; and 95% CI: −2.38 to 7.05). Similarly, Week 2 did not differ significantly from Week 3 (*p* = 0.79; d = 0.46; and 95% CI: −4.32 to 3.25) or Week 4 (*p* = 0.58; d = 0.38; and 95% CI: −1.30 to 4.90). Finally, no significant difference was observed between Week 3 and Week 4 (*p* = 1.00; d = 0.08; and 95% CI: −6.22 to 1.96) (Table 3).

#### 3.2.2. External Training Load

The statistical analysis showed that TD (F(1,14) 1.767, *p* = 0.205, and pη^2^ = 0.112) did not report a significant effect of time. Pairwise comparisons revealed no significant differences between Week 1 and Week 2 (*p* = 1.00; d = 0.09; and 95% CI: −269.51 to 163.44), Week 3 (*p* = 1.00; d = 0.22; and 95% CI: −494.14 to 283.40), or Week 4 (*p* = 1.00; d = 0.20; and 95% CI: −306.58 to 115.29). Similarly, Week 2 did not differ significantly from Week 3 (*p* = 1.000; d = 0.09; and 95% CI: −560.32 to 455.66) or Week 4 (*p* = 1.000; d = 0.08; and 95% CI: −288.65 to 203.16). Finally, no significant difference was observed between Week 3 and Week 4 (*p* = 1.00; d = 0.02; and 95% CI: −413.96 to 433.12) (Table 4). The statistical analysis revealed that DMS (F(1,14)8.384, *p* = 0.475, and pη^2^ = 0.375) did not report a significant effect of time. Pairwise comparisons revealed no significant differences between Week 1 and Week 2 (*p* = 1.00; d = 0.09; and 95% CI: −56.81 to 36.74), Week 3 (*p* = 0.36; d = 0.33; and 95% CI: −88.40 to 17.74), or Week 4 (*p* = 0.11; d = 0.22; and 95% CI: −52.89 to 3.69). Similarly, Week 2 did not differ significantly from Week 3 (*p* = 0.06; d = 0.24; and 95% CI: −51.49 to 0.89) or Week 4 (*p* = 1.00; d = 0.13; and 95% CI: −61.75 to 32.62). Finally, no significant difference was observed between Week 3 and Week 4 (*p* = 1.00; d = 0.11; and 95% CI: −31.77 to 53.24) (Table 4).

A repeated-measures ANOVA showed a significant main effect of time on the DHS (F(1,14) = 19.552, *p* = 0.001, ηp^2^ = 0.583). Pairwise comparisons indicated that Week 1 values were significantly lower than Week 3 (*p* = 0.0001; d = 0.94; and 95% CI: −58.19 to −17.97) and Week 4 (*p* = 0.02; d = 0.77; and 95% CI: −85.20 to 7.72), respectively, while Week 2 values were likewise lower than Week 3 (*p* = 0.001; d = 1.17; and 95% CI: −58.28 to −14.84) and Week 4 (*p* = 0.010; d = 0.95; and 95% CI: −80.19 to −7.72) (Table 4).

There was a significant main effect of time on DACC (F(1,14) = 11.237, *p* = 0.005, and ηp^2^ = 0.445). Post hoc tests indicated that Week 3 and Week 4 values were significantly higher than Week 1 (*p* = 0.03; d = 0.83; and 95% CI: −16.13 to −0.83 and *p* = 0.02; d = 0.65; and 95% CI: −14.02 to 0.29, respectively), and Week 2 data was significantly lower than Week 3 (*p* = 0.02; d = 0.78; and 95% CI: −15.00 to −1.16). Furthermore, no significant differences were observed between Week 2 and Week 1 (*p* = 1.00; d = 0.02; and 95% CI: −3.11 to 3.90) or Week 4 (*p* = 0.06; d = 0.64; and 95% CI: −13.2 to 0.26) (Table 4).

Similarly, DDEC showed a significant main effect of time (F(1,14) = 12.750, *p* = 0.003, and ηp^2^ = 0.447). Post hoc tests indicated that Week 3 and Week 4 data were significantly greater than Week 1 (*p* = 0.03; d = 0.62; and 95% CI: −15.88 to −0.65 and *p* = 0.04; d = 0.55; and 95% CI: −13.74 to −0.82, respectively), and the Week 2 value was significantly lower than Week 3 and Week 4 (*p* = 0.02; d = 1.19; and 95% CI: −14.24 to −0.36 and *p* = 0.03; d = 1.11; and 95% CI: −11.99 to −0.64, respectively). In addition, no significant difference was observed between Week 1 and Week 2 (*p* = 1.00; d = 0.48; and 95% CI: −4.75 to 2.82) (Table 4).

**Table 4:** External training load parameters measured during the 4 weeks of the experimental protocol.

Parameters	Week 1	Week 2	Week 3	Week 4
**TD** **(m)**	5911.3 ± 436.8	5964.4 ± 611.4	6016.7 ± 581.8	6007.1 ± 509.7
	95% CI:	95% CI:	95% CI:	95% CI:
	5669.4–6153.2	5625.8–6309.9	5694.5–6338.9	5724.8–6289.4
**DMS** **(m)**	422.1 ± 111.7	432.2 ± 105.7	457.5 ± 99.6	446.7 ± 106.7
	95% CI:	95% CI:	95% CI:	95% CI:
	360.2–484.0	373.6–490.7	402.3–512.6	387.6–505.8
**DHS** **(m)**	51.2 ± 26.5 ^c,e^	42.9 ± 30.5 ^b,d^	79.3 ± 33.3	87.7 ± 60.9
	95% CI:	95% CI:	95% CI:	95% CI:
	44.7–55.8	44.6–56.7	60.8–97.8	53.9–121.4
**DACC** **(m)**	50.2 ± 9.9 ^a,e^	50.6 ± 10.9 ^b^	58.7 ± 11.4	57.1 ± 11.5
	95% CI:	95% CI:	95% CI:	95% CI:
	44.7–55.8	44.6–56.7	52.4–65.1	50.7–63.5
**DDEC** **(m)**	51.2 ± 15.4 ^a,d^	44.5 ± 13.9 ^b,d^	59.5 ± 12.3	58.5 ± 12.2
	95% CI:	95% CI:	95% CI:	95% CI:
	42.7–59.7	44.5–59.9	52.6–66.3	51.7–65.3

TD, total distance; DMS, distance at moderate speed; DHS, distance at high speed; DACC, distance traveled in acceleration; and DACC, distance traveled in deceleration. ^a^ Significantly different from 3° week for *p* < 0.05; ^b^ significantly different from 3° week for *p* < 0.01; ^c^ significantly different from 3° week for *p* < 0.001; ^d^ significantly different from 4° week for *p* < 0.05; and ^e^ significantly different from 4° week for *p* < 0.01.

#### 3.2.3. Correlations

**Figure 6:**
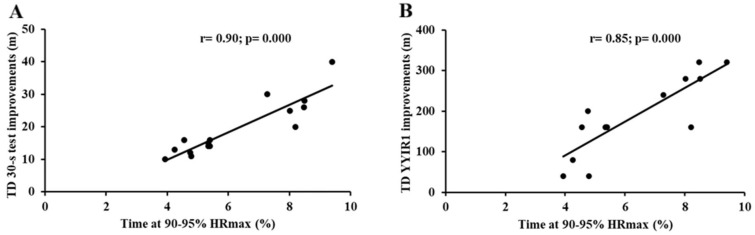
Relationship between (**A**) TD improvements in 30-s test and time spent at 90–95% of maximal heart rate (HRmax); (**B**) TD improvements in YYIR1 and time spent at 90–95% of maximal heart rate (HRmax). Abbreviations: 30 s test, 30-s test; YYIR1, Yo-Yo Intermittent Recovery Test Level 1.

A very large correlation between the training time spent at 90–95% HRmax and total distance improvement in the 30-s test (r = 0.90, 95% CI: 0.87–0.98, and *p* = 0.0001; Figure 6A) was observed. A nearly perfect correlation between the same exposure and TD improvements during YYIR1 (r = 0.81, 95% CI: 0.63–0.97, and *p* = 0.0001; Figure 6B) was also found.

**Figure 7:**
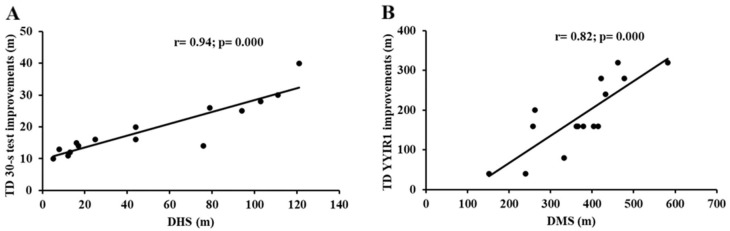
Relationship between (**A**) improvements of TD in 30-s test and distance at high speed; (**B**) improvements of TD in YYIR1 and distance at moderate speed. Abbreviations: 30-s test, 30-s test; YYIR1, Yo-Yo Intermittent Recovery Test Level 1; DHS, distance at high speed; and DMS, distance at moderate speed.

DHS showed a very large correlation with the improved TD in the 30-s test (r = 0.94, 95% CI: 0.87–0.98, and *p* = 0.0001; Figure 7A), as well as with gains in the TD during YYIR1 (r = 0.82, 95% CI:0.57–0.94, and *p* = 0.0001; Figure 7B).

**Figure 8:**
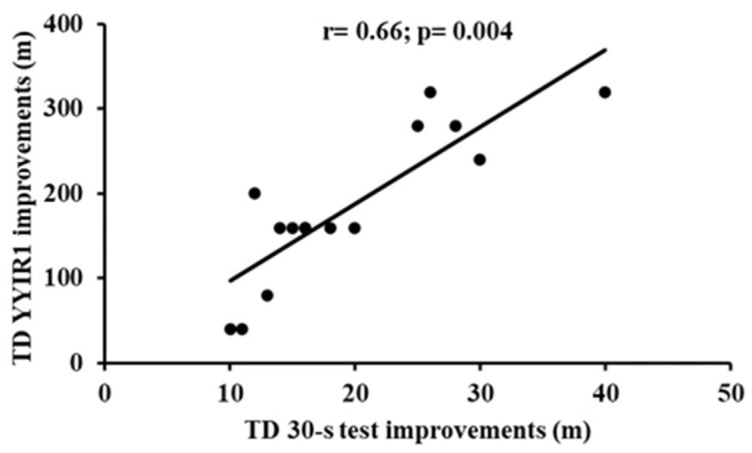
Relationship between TD improvements in 30-s test and in YYIR1. Abbreviations: 30-s test, 30-second test; YYIR1, Yo-Yo Intermittent Recovery Test Level 1.

Finally, a significant positive correlation between TD changes in the 30-s test and TD improvements in the YYIR1 test (r = 0.66, 95% CI: 0.32–0.86, and *p* = 0.004; Figure 8) was found.

## 4. Discussion

This pilot study examined the effects of an off-season HIIT program combining Small-Sided Games (SSGs) and Repeated Sprint Training (RST) on aerobic performance and sprint endurance ability in U-15 female football players.

### 4.1. Effects of Training

The main finding of our study was that, after the 4-week training period, both performance outcomes showed significant improvements. Specifically, sprint endurance ability, as indicated by the total distance covered during the 30-second sprint test, increased substantially. Similarly, aerobic performance, assessed through the total distance completed in the Yo-Yo Intermittent Recovery Level 1 (YYIR1) test, also demonstrated a marked enhancement.

Our findings are in line with those reported by Stankovic et al. (2023) [14], showing that various HIIT protocols produce significant improvements in both aerobic and anaerobic performance in young female team athletes. This result indicates that female adolescent football players can achieve substantial adaptations following a high-intensity interval training program. This is consistent with previous recent systematic reviews [14,33] reporting that female youth athletes can attain relative improvements comparable to those of their male peers when training load and recovery are properly managed.

Players’ aerobic performance levels significantly impact match performance, influencing the total distance covered, the number of sprints performed, ball involvement, and overall technical and tactical execution [27]. Research showed that an 8–12% improvement in aerobic performance correlates with enhanced football-specific activities without compromising neuromuscular performance [13,34].

In the present study, adolescent female football players increased their YYIR1 performance by approximately 20%. This improvement is consistent with findings from Hill-Hass et al. (2009) [35], who reported similar gains in adolescent male football players. Moreover, our results align with those of Impellizzeri et al. (2006) [13], who reported that both SSGs and HIIT produce significant improvements in aerobic performance in male adolescent football players. These findings support the use of both training modalities as effective strategies for enhancing aerobic performance within this population. It is worth noting that the magnitude of improvement observed in our female sample exceeds what was typically reported in the aforementioned studies on male adolescent players [13,35]. As demonstrated in previous studies [36,37], the differences in maturation status, hormonal profile, and cardiac dimensions may have modulated the adaptations to HIIT.

Our results are in agreement with those of Iaia et al. (2009) [26], who found that various HIIT protocols can elicit positive adaptations in aerobic performance. Similarly, Ferrari-Bravo et al. (2007) [38] observed improvements of 22% and 17% in YYIR1 performance in young football players who underwent a running HIIT program during the pre-season. Taken together, our finding supports the growing body of evidence suggesting that young female athletes respond favorably to HIIT and provides the efficacy of a training program considering recovery demands and maturation-related variability.

In relation to the significant improvement in sprint endurance observed in our sample, scientific evidence reported the effectiveness of HIIT-based training in stimulating aerobic and anaerobic adaptations in female football players [14,39]. These studies demonstrated that HIIT formats, such as SSGs and RST, are able to impart beneficial effects on sprint endurance in this specific population. Such improvements can be attributed to repeated activation of the ATP–phosphocreatine (ATP-PCr) system and anaerobic glycolysis during successive near-maximal bouts with brief recoveries, thereby enhancing glycolytic enzyme activity and neuromuscular adaptations that support high-intensity performance [40].

Specifically, in female football players, sport-specific HIIT protocols proved to produce significant gains in both aerobic and anaerobic capacities. Randers et al. (2013) [41] reported increases in the VO_2_max and intermittent endurance after 16 weeks of training, and a systematic review similarly observed performance benefits in female team-sport athletes [14]. HIIT also upregulates the peroxisome proliferator-activated receptor-gamma coactivator-1α (PGC-1α), a key regulator of mitochondrial biogenesis and oxidative metabolism, thus improving metabolic efficiency and fatigue resistance during repeated sprints [42,43].

In regards to physiological and metabolic responses, post-exercise blood lactate concentration ([La]^+^) measured at the end of the 30-s test did not show significant changes despite a clear improvement in TD performance. This result may be partly explained by the close similarity in the exercise modalities of RST and the 30-s test, both characterized by short-duration and high-intensity shuttle running efforts. Such specificity likely promoted targeted physiological adaptations that improved performance without increasing metabolic strain, as reflected by stable lactate values. Conversely, the extended duration of the YYIR1 test resulted in greater metabolic stress, as evidenced by the significant increase in the post-exercise ([La]^+^) value.

Additionally, the HRmax values recorded in this study, during both the 30-s test and YYIR1, were comparable to those reported in amateur and adult female football players [16,24].

The internal workload, assessed during training sessions using peak heart rate (HRpeak) and mean heart rate (HRmean), showed no significant differences across the weeks. This consistency could be attributed to the strong encouragement given to all players to exert maximum effort during the exercise. Moreover, during the intervention period, participants spent 7% of the total training time at >85% of their HRmax, a result closely aligned with previous studies in male football players [13,44,45], which reported that approximately 8% of training time was spent at high intensity. This finding reinforces the idea that even a relatively short training period (4 weeks) can effectively enhance aerobic performance and sprint endurance ability in adolescent female football players during the off-season.

The very large correlations between TD changes in the 30-s test and in the YYIR1 and the time accumulated at the 90–95% HRmax underscore the key role of high-intensity exposure in improving aerobic performance and sprint endurance. Training in this zone is known to increase the maximal oxygen uptake (VO_2_max) and, in some contexts, running economy [46]. It also enhances the repeated sprint ability, consistent with performance gains following high-intensity interval training [11]. These results align with Laursen and Buchheit (2019) [11], who reported concurrent improvements in aerobic performance and sprint endurance with similar protocols. Accordingly, Small-Sided Games and repeated sprint training during the off-season are likely to induce favorable aerobic and anaerobic adaptations in youth football [47,48].

This study also explored the relationship between improvements in aerobic performance and sprint endurance and the external load parameters recorded during training sessions. A very large correlation between distance at high speed (DHS) and sprint endurance improvements were observed, thus indicating that players who covered greater distances at a high speed experienced the most significant improvements in sprint endurance. Our findings underscore the principle of anaerobic training specificity, whereby performance adaptations are optimized when the training stimulus closely mirrors the metabolic and mechanical demands of competition. In particular, high-speed running elicits substantial activation of the glycolytic and phosphagen energy systems, thereby inducing specific neuromuscular and metabolic adaptations that are critical for enhancing sprint endurance.

The observed strong association between the DHS and improvements in sprint performance suggests that both the volume and intensity of high-speed efforts represent key determinants of anaerobic conditioning. Accordingly, the systematic monitoring and manipulation of DHS during training may serve as an effective strategy to elicit targeted adaptations in line with the repeated high intensity demands of match play.

Furthermore, our results showed a very strong correlation between improvements in the moderate speed distance (DMS) and total distance (TD) in YYIR1. Players covering a greater DMS achieved the most significant gains in aerobic performance, confirming the YYIR1 test’s validity for football players and aligning with previous research [49,50,51]. This relationship reflects the aerobic training specificity of SSG, which emphasizes intermittent moderate-intensity efforts that stimulate cardiovascular and metabolic adaptations relevant to match demands. Increased DMS during SSG likely drives improvements in oxidative capacity and recovery ability, key factors measured by YYIR1.

Therefore, monitoring DMS in training can help coaches tailor programs to effectively enhance aerobic performance, particularly during the off-season, reinforcing the role of SSGs in sport-specific endurance development.

Finally, the moderate correlation observed between TD improvements in the 30-s test and in the YYIR1 test suggests that enhancements in short-duration, high-intensity efforts may contribute to better intermittent endurance. This finding also indicates that training interventions targeting anaerobic power development could also support performance in repeated sprint or high-intensity intermittent effort capacities that are particularly relevant in team sports contexts.

### 4.2. Limitations and Future Directions

This pilot study has several limitations. First, the reduced small sample size may have led to the possibility of statistically underpowered results, thus limiting the generalizability of data.

Second, the absence of a control group limits the generalizability of our results, and, as such, this investigation should be regarded primarily as a case study.

Third, the relatively short duration of the intervention period may have limited the extent of the observable outcomes. Future studies should consider longer experimental training programs to capture more pronounced physiological and performance-related changes.

Finally, the menstrual cycle was not monitored. The absence of menstrual monitoring did not allow us to evaluate the influence of the menstrual cycle on performance and training responses.

### 4.3. Practical Applications

This study demonstrates that a 4-week off-season program combining SSG and RST twice a week can effectively improve the aerobic performance level as well as sprint endurance ability in Under-15 female football players. Therefore, football coaches can implement, within the weekly training program, two sessions of SSGs and two sessions of RST to enhance aerobic fitness without compromising sprint performance. Moreover, a single session including a structured warm-up, technical drills, and SSG or RST protocols, followed by a brief cool-down, is recommended. Additionally, monitoring training intensity through heart rate and movement metrics allows for the individualization of the internal and external training load, thus helping to maintain fitness, reduce early-season injury risk, and ensure players enter the pre-season in optimal condition.

## 5. Conclusions

This study is the first to investigate the impact of a HIIT program on off-season detraining in adolescent female football players. Our study highlights the efficacy of weekly HIIT-based training combining SSG and RST in enhancing both the aerobic capacity and sprint endurance ability, thus minimizing performance declines during the off-season. Therefore, in U-15 female football players, a 4-week combined HIIT program during the off-season is recommended to start the pre-season with a satisfactory level of aerobic fitness and sprint endurance.

## Figures and Tables

**Table 1:** Weekly training program description.

Time Period	Tuesday	Wednesday	Thursday	Friday
*WEEK 1–2*	10 min of technical drills 25 min of SSGs	10 min of technical drills 3 sets of 5 reps RST Rest: 20 s between repetition and 4 min between sets	10 min of technical drills 25 min SSGs	10 min of technical drills 3 sets of 5 reps RST Rest: 20 s between repetition and 4 min between sets
*WEEK 3–4*	10 min of technical drills 25 min of SSGs	10 min of technical drills 3 sets of 6 reps RST Rest: 20 s between repetition and 4 min between sets	10 min of technical drills 25 min of SSGs	10 min of technical drills 3 sets of 6 reps RST Rest: 20 s between repetition and 4 min between sets

SSGs, Small-Sided Games; RST, Repeated Sprint Training.

**Table 2:** Changes from pre- to post-tests.

Parameters		30 s Test			YYIR1	
	Pre	Post	Statistics	Pre	Post	Statistics
**TD** **(m)**	155.3 ± 12.5 95% CI: 161.3–172.1	175.3 ± 8.3 *** 95% CI: 170.3–180.4	t(14) = 3.389, *p* = 0.004, d = 0.91	485.3 ± 130.8 95% CI: 412.9–557.8	658.7 ± 157.3 *** 95% CI: 562.1–749.9	t(14) = 5.208, *p* = 0.0001, d = 1.12
**HRpeak** **(bpm)**	195.7 ± 4.6 95% CI: 193.1–198.2	196.2 ± 4.3 95% CI: 193.8–198.6	t(14) = 1.293, *p* = 0.217, d = 0.27	195.1 ± 5.1 95% CI: 192.3–197.9	197.1 ± 5.1 95% CI: 194.2–199.9	t(14) = 1.260, *p* = 0.127, d = 0.38
**[LA]^+^** **(mmol·L^−1^)**	11.9 ± 4.2 95% CI: 10.7–12.9	12.1 ± 1.1 95% CI: 11.5–12.7	t(14) = 0.578, *p* = 0.572, d = 0.14	9.9 ± 0.8 95% CI: 9.5–10.4	11.4 ± 1.2 *** 95% CI: 10.4–12.1	t(14) = 5.367, *p* = 0.0001, d = 1.37

TD, total distance; HRpeak, heart rate peak; [La]^+^, blood lactate concentration; YYIR1, Yo-Yo Intermittent Recovery Test Level 1; 30-s test, 30-second test. *** *p* < 0.001.

**Table 3:** The internal training load parameters measured the 4 weeks of the experimental protocol.

Parameters	Week 1	Week 2	Week 3	Week 4
**HRpeak** **(bpm)**	192.1 ± 2.1	194.3 ± 3.3	195.6 ± 2.6	196.1 ± 3.6
	95% CI:	95% CI:	95% CI:	95% CI:
	190.9–193.3	192.5–196.2	194.2–197.0	194.1–198.1
**HRmean** **(bpm)**	140.6 ± 4.9	140.1 ± 4.6	137.9 ± 4.9	138.3 ± 4.8
	95% CI:	95% CI:	95% CI:	95% CI:
	137.9–143.3	137.5–142.6	135.2–140.6	135.6–140.9

HRpeak, heart rate peak; HRmean, heart rate mean.

## Data Availability

Data generated or analyzed during this study are available from the corresponding author upon reasonable request.
